# Do patients with ischaemic cardiomyopathy benefit from off-pump coronary bypass surgery? (From the KROK registry)

**DOI:** 10.1093/icvts/ivaf014

**Published:** 2025-02-06

**Authors:** Kinga Kosiorowska, Marek Jasiński, Roman Przybylski, Marek Deja, Jan Rogowski, Witold Gerber, Jerzy Pacholewicz, Romuald Cichoń, Marek Cisowski, Wojciech Pawliszak, Paweł Bugajski, Michał Krejca, Tomasz Hirnle, Bartłomiej Perek, Zdzisław Tobota, Bohdan Maruszewski, Tomasz Hrapkowicz, KROK Investigators, W Gryszko, K Jarmoszewicz, W Pawliszak, J Rogowski, P Suwalski, P Zelazny, K Kusmierski, T Hirnle, M Jamielity, M Kusmierczyk, K Wróbel, R Stanislawski, R Gocol, P Bugajski, W Gerber, K Widenka, B Kapelak, J Pacholewicz, M Krejca, G Religa, K Karpeta, E Pietrzyk, L Tulecki, M Burysz, J Skiba, M Cisowski, W Szczawinski, J Stazka

**Affiliations:** Department of Cardiac Surgery and Heart Transplantation, Institute of Heart Diseases, Wroclaw Medical University, Wroclaw, Poland; Department of Cardiac Surgery and Heart Transplantation, Institute of Heart Diseases, Wroclaw Medical University, Wroclaw, Poland; Department of Cardiac Surgery and Heart Transplantation, Institute of Heart Diseases, Wroclaw Medical University, Wroclaw, Poland; Department of Cardiac Surgery, Medical University of Silesia, Katowice, Poland; Department of Cardiac and Vascular Surgery, Medical University of Gdansk, Gdansk, Poland; Department of Cardiac Surgery, American Heart of Poland, Bielsko-Biała, Poland; Department of Cardiac Surgery, Pomeranian Medical University, Szczecin, Poland; Department of Cardiac Surgery, MEDINET Heart Center Ltd, Wroclaw, Poland; Department of Cardiac Surgery, University Hospital, Institute of Medical Sciences, University of Opole, Opole, Poland; Department of Cardiac Surgery, Nicolaus Copernicus University in Toruń, Ludwik Rydygier Collegium Medicum in Bydgoszcz, Bydgoszcz, Poland; Department of Cardiac Surgery, J. Strus Hospital, Poznan, Poland; Department of Cardiac Surgery, Medical University of Lodz, Lodz, Poland; Department of Cardiac Surgery, Medical University of Bialystok, Bialystok, Poland; Department of Cardiac Surgery and Transplantology, Poznan University of Medical Sciences, Poznan, Poland; Department of Pediatric Cardiothoracic Surgery, The Children’s Memorial Health Institute, Warsaw, Poland; Department of Pediatric Cardiothoracic Surgery, The Children’s Memorial Health Institute, Warsaw, Poland; Department of Cardiac, Vascular and Endovascular Surgery and Transplantology, Medical University of Silesia, Zabrze, Poland

**Keywords:** left ventricle dysfunction, ischaemic cardiomyopathy, off-pump coronary artery bypass grafting, surgical revascularization

## Abstract

**OBJECTIVES:**

This study aimed to compare perioperative outcomes and long-term mortality between off-pump coronary artery bypass grafting and on-pump coronary artery bypass grafting in patients with ischaemic cardiomyopathy who had a left ventricle ejection fraction of ≤35%.

**METHODS:**

A retrospective cohort analysis was conducted using data from the Polish National Registry of Cardiac Surgery Procedures database, encompassing patients who underwent isolated coronary artery bypass grafting in Poland between 2012 and 2022. Patients were divided into two groups: on-pump and off-pump. Propensity score matching was used to balance the groups. The primary outcome was long-term all-cause mortality following surgical revascularization.

**RESULTS:**

A total of 9920 patients were included, with 3116 patients in each group after propensity score matching. The median follow-up period was 4 years. The off-pump group was associated with a lower 30-day mortality rate (6.4% vs 9.1%, *P* = 0.002) and fewer perioperative complications. However, long-term survival analysis revealed a modest but statistically significant advantage for on-pump group at the 10-year follow-up (*P* = 0.047).

**CONCLUSIONS:**

Off-pump provides short-term benefits, including reduced early mortality and fewer complications compared to on-pump technique. However, these advantages do not translate into improved long-term survival, where on-pump demonstrates a slight benefit. The choice between off-pump and on-pump technique should be individualized based on patient-specific factors and surgical expertise.

## GRAPHICAL ABSTRACT

**Figure:**
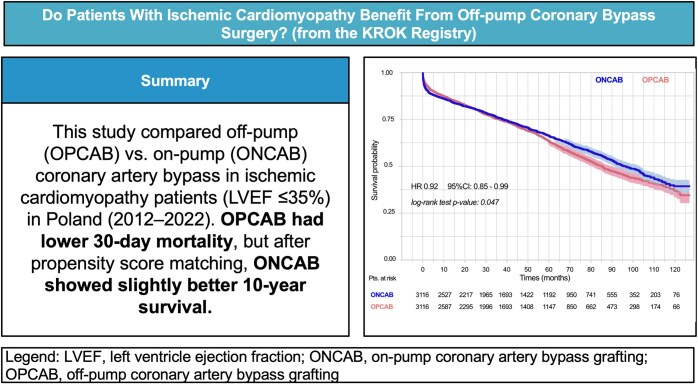

## INTRODUCTION

Ischaemic cardiomyopathy (ICM) manifests as reduced left ventricle ejection fraction (LVEF) due to acute or acute on chronic coronary artery disease, posing a significant global health challenge [1]. Treatment strategies aim to improve coronary blood flow, relieve symptoms and prevent irreversible myocardial damage. Options include medical therapy, percutaneous coronary intervention (PCI) and coronary artery bypass grafting (CABG), the latter being the gold standard for selected patients [1–3]. Untreated, ICM progresses to heart failure and death. Heart transplantation is considered the ultimate treatment for end-stage ICM but is limited by donor scarcity. Ventricular assist devices offer temporary or long-term support but have significant drawbacks, including high costs, potential complications such as driveline infections and the need for lifelong anticoagulation therapy, making this option challenging for many patients [1]. The landmark STICH trial evaluated various surgical strategies in ICM patients, demonstrating that despite higher initial risks, CABG offers acceptable early mortality rates and long-term benefits, particularly in those with lower LVEF and markedly dilated left ventricle [2, 4–6]. The STICH Extended Study revealed that combining CABG with pharmacological therapy reduces all-cause mortality by 16% compared to optimal medical therapy alone [7]. Notably, CABG in patients with left ventricular failure not only yields better outcomes than conservative treatment but may also reduce all-cause and cardiac-related mortality and the need for repeat revascularization compared to PCI with drug-eluting stents [8]. The choice between off-pump coronary artery bypass grafting (OPCAB) and on-pump coronary artery bypass grafting (ONCAB) techniques is pivotal in ICM patients. Despite initial enthusiasm, OPCAB’s appeal has waned, particularly in low-risk patients [9, 10]. Nevertheless, valid indications persist, suggesting potential benefit in high-risk scenarios.

This study compares OPCAB and ONCAB techniques in high-risk ICM patients, evaluating their perioperative outcomes and impact on long-term mortality.

## MATERIALS AND METHODS

### Data source

This retrospective, multicentre cohort study used data from the Polish National Registry of Cardiac Surgery Procedures (KROK) for patients undergoing isolated CABG at cardiac surgery centres in Poland. The registry serves as a comprehensive repository for all cardiac surgeries conducted across Poland. It mandates data transfer from participating centres under the agreement with the Polish Ministry of Health. The registry includes crucial information on patients’ preoperative status, risk factors, operative techniques, perioperative course and early outcomes. Long-term all-cause mortality data were obtained by linking to the National Health System records, ensuring comprehensive follow-up. To maintain strict confidentiality, all data were blinded, preventing the identification of patients and their respective treatment centres. This approach aligns with the highest standards of data protection and ethical research conduct. The study was performed in accordance with the ethical principles outlined in the Declaration of Helsinki. The institutional bioethics committee reviewed the study and officially exempted it from further ethical review due to its retrospective nature and the use of anonymized data from the national registry. Consequently, the requirement for patient consent was waived. Furthermore, the study protocol was found to adhere to all applicable ethical standards for scientific research, reinforcing the integrity of the research methodology and the reliability of the findings.

### Study population

**Figure 1:**
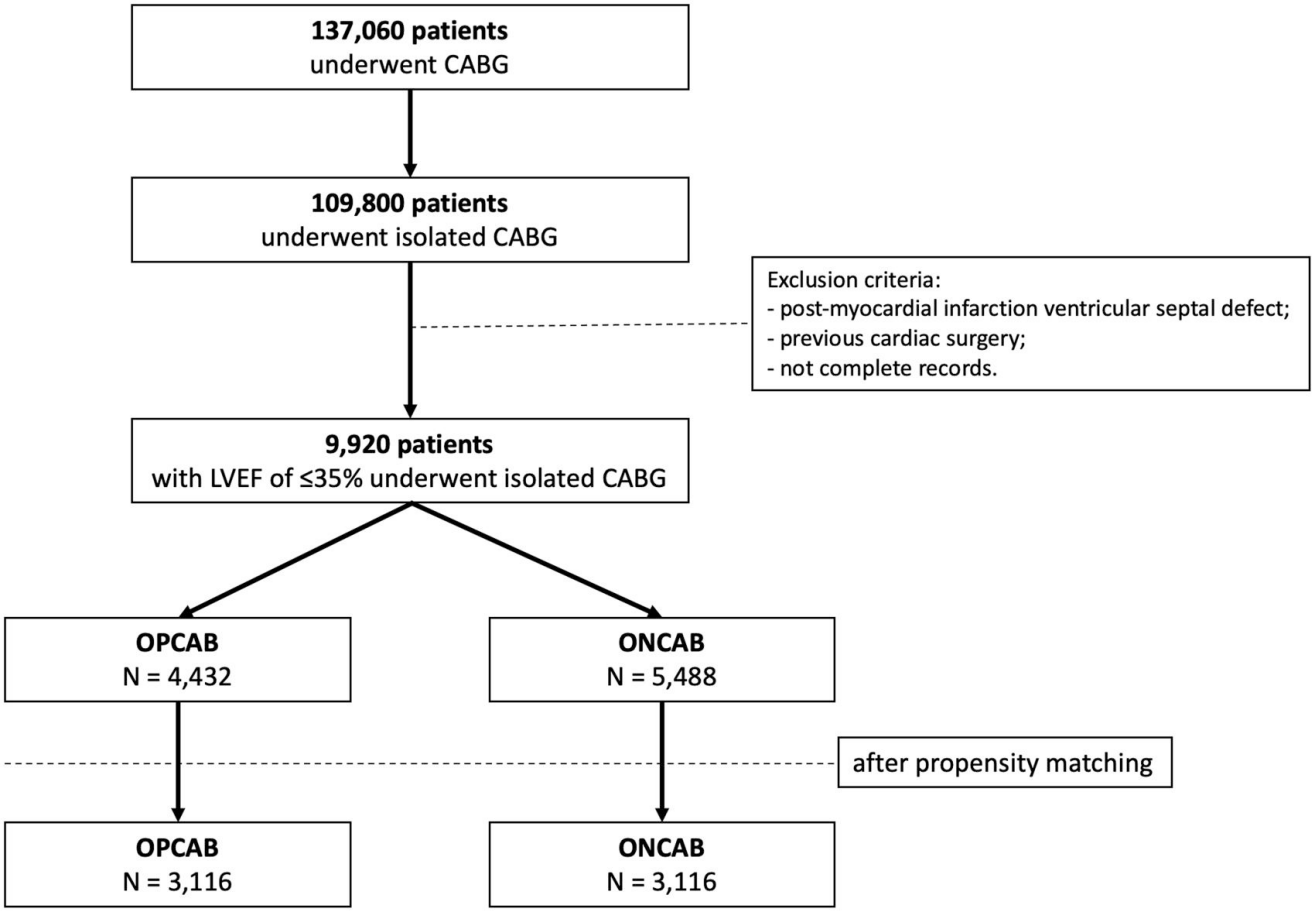
Flowchart of the study cohort

The study included patients who underwent primary isolated CABG between January 2012 and July 2022, with preoperative LVEF of ≤35%. Exclusions were: (i) post-myocardial infarction ventricular septal defect, (ii) previous cardiac surgery and (iii) incomplete records. Patients were divided into two groups based on the surgical technique employed: ONCAB and OPCAB. In order to avoid selection bias, we conducted the analysis according to the ‘intention-to-treat’ principle. Patients who were initially assigned to the OPCAB group but were later converted to ONCAB were still analysed within the OPCAB group, preserving the integrity of the original treatment assignment. A flowchart illustrating the study cohort is shown in Fig. 1.

In our study, the term ‘critical preoperative state’ is defined as a condition in which patients require immediate and significant medical interventions prior to undergoing surgery. These interventions may include the administration of intravenous heparin, nitroglycerin, intra-aortic balloon pump (IABP) support or inotropes. Patients categorized as being in a critical preoperative state are typically unstable and may present with life-threatening conditions, necessitating urgent or emergent care to stabilize their condition before revascularization.

This definition distinguishes ‘critical preoperative state’ from ‘urgent revascularization’, which involves procedures performed during the same hospital admission but does not necessarily indicate a life-threatening condition. While patients in urgent revascularization may receive intravenous heparin or nitroglycerin as part of their management, they do not require intensive preoperative interventions such as IABP or inotropes. The patients identified within the ‘critical preoperative state’ category were at a higher risk, as the need for such intensive measures signifies a more severe level of instability.

### Study end-points

The primary end-point was long-term all-cause mortality. Secondary end-points included the assessment of grafts performed, postoperative complications, as well as the intensive care unit (ICU) and hospital length of stay. Long-term survival was analysed and compared between the groups without differentiating specific causes of death.

### Statistical analysis

Summary statistics were calculated to describe the data, with continuous variables reported as means ± standard deviations and categorical variables expressed as counts and percentages. For cases from the KROK database with up to 5% missing data, the multiple imputation by chained equations (MICE) algorithm was applied to handle missing values. Covariate balance was assessed using standardized mean differences (SMD), with an SMD <0.1 considered indicative of good balance between groups. Overlap assessment involved visually inspecting the propensity score distributions of the ONCAB and OPCAB groups, confirming sufficient common support. Statistical analyses were conducted using the Chi-squared test for qualitative variables and Student’s t-test for quantitative variables, whereas analyses on the matched data using paired tests to account for the dependency introduced by matching. Univariate logistic regression was conducted on both the entire cohort and the matched sample. Kaplan–Meier analysis was used to evaluate long-term survival, with the proportional hazards assumption assessed using scaled Schoenfeld residuals. A two-sided *P*-value <0.05 was considered statistically significant. All analyses were conducted using R software within the RStudio environment.

## RESULTS

A total of 137 060 coronary artery disease surgeries were reported during the study period, with 109 800 patients undergoing isolated CABG. Of these, 9920 patients with a preoperative LVEF of ≤35% were included in the final analysis, of which 44.7% underwent OPCAB. In the OPCAB group, 110 patients (2.5%) required conversion to ONCAB, and the 30-day mortality rate for those converted patients was 25.5%. Detailed preoperative and postoperative characteristics of the entire cohort are presented in Table 1.

**Table 1:** Baseline characteristics of patients according to operative technique

Clinical variables	All patients					Propensity-matched patients				
	ONCAB (*n* = 5488)	OPCAB (*n* = 4432)	SMD	95% CI	*P*-value	ONCAB (*n* = 3116)	OPCAB (*n* = 3116)	SMD	95% CI	*P*-value
Age, years	65.93 (8.52)	66.24 (8.66)	0.04	−0.01, 0.08	0.078	66.23 (8.53)	66.26 (8.57)	<0.01	−0.05, 0.05	0.89
Male	4611 (84.0)	3573 (83.6)	0.01	−0.03, 0.05	0.57	496 (15.9)	504 (16.2)	−0.01	−0.06, 0.04	0.81
Body mass index	27.85 (4.45)	27.81 (4.52)	−0.02	−0.06, 0.02	0.74	27.74 (4.49)	27.7 (4.49)	<0.01	−0.05, 0.05	0.93
EuroSCORE	4.94 (7.11)	4.73 (6.54)	−0.02	−0.06, 0.02	0.124	4.89 (6.61)	4.83 (6.39)	0.01	−0.04, 0.06	0.74
LVEF	29.84 (5.03)	29.91 (5.01)	0.01	−0.03, 0.05	0.48	29.88 (4.99)	29.93 (5.03)	−0.01	−0.06, 0.04	0.67
CCS III	1692 (30.9)	16690 (39.2)	0.17	0.13, 0.21	<0.001	1135 (36.4)	1157 (37.1)	−0.01	−0.06, 0.03	0.56
CCS IV	1125 (20.6)	867 (20.4)	0.01	−0.03, 0.05	<0.001	659 (21.1)	653 (21.0)	0.01	−0.04, 0.05	0.85
Recent myocardial infarction (within 30 days)	2653 (48.3)	1866 (43.6)	−0.04	−0.08, 0.01	<0.001	1479 (47.5)	1490 (47.8)	−0.07	−0.57, 0.43	0.80
Diseased major coronary arteries	2.76 (0.5)	2.46 (0.73)	−0.39	−0.43, −0.35	<0.001	2.70 (0.52)	2.70 (0.55)	<0.01	−0.05, 0.05	1.00
Left main stem > 50%	2099 (38.2)	1369 (32.0)	−0.08	−0.12, −0.04	<0.001	1200 (38.5)	1178 (37.8)	0.01	−0.04, 0.06	0.58
Preoperative condition										
Inotropic support	432 (7.9)	221 (5.2)	−0.10	−0.14, −0.06	<0.001	185 (5.9)	182 (5.8)	<0.01	−0.05, 0.05	0.91
Intravenous nitroglycerin/heparin	972 (17.7)	612 (14.3)	−0.08	−0.12, −0.04	<0.001	522 (16.8)	520 (16.7)	<0.01	−0.05, 0.05	0.97
Preoperative IABP	321 (5.8)	106 (2.5)	−0.16	−0.19, −0.12	<0.001	75 (2.4)	77 (2.5)	<0.01	−0.05, 0.05	0.94
Cardiogenic shock	230 (4.2)	137 (3.2)	−0.04	−0.08, −0.01	0.013	78 (2.5)	78 (2.5)	<0.01	−0.05, 0.05	0.94
Critical preoperative state	380 (6.9)	205 (4.8)	−0.08	−0.12, −0.04	0.000	163 (5.2)	148 (4.7)	0.02	−0.03, 0.07	0.42
Mechanical ventilation	97 (1.8)	46 (1.1)	−0.05	−0.09, −0.01	0.006	48 (1.5)	41 (1.3)	0.02	−0.03, 0.07	0.52
Comorbidities										
Renal insufficiency	609 (1.1)	553 (12.9)	0.05	0.01, 0.09	0.006	438 (14.1)	443 (14.2)	<0.01	−0.05, 0.05	0.88
Smoking	3685 (67.1)	2984 (69.8)	0.06	0.02, 0.10	0.006	2341 (75.1)	2343 (75.2)	<0.01	−0.05, 0.05	0.98
Chronic lung disease	593 (10.8)	457 (10.7)	−0.01	−0.05, 0.03	0.88	379 (12.2)	361 (11.6)	0.02	−0.03, 0.07	0.51
Arterial hypertension	4733 (86.2)	3429 (80.2)	−0.16	−0.20, −0.12	<0.001	2711 (87.0)	2731 (87.6)	−0.02	−0.07, 0.03	0.47
Pulmonary hypertension	579 (10.5)	508 (11.9)	0.05	0.01, 0.09	0.041	408 (13.1)	403 (12.9)	0.01	−0.04, 0.06	0.8
Hyperlipidaemia	3745 (68.2)	2741 (64.1)	−0.11	−0.13, −0.05	<0.001	2163 (69.4)	2166 (69.5)	<0.01	−0.05, 0.05	0.96
Diabetes	2184 (39.8)	1798 (42.0)	0.05	0.01, 0.09	0.025	643 (20.6)	656 (21.1)	−0.06	−0.11, −0.01	0.71
Peripheral artery disease	1414 (25.8)	924 (21.6)	−0.11	−0.15, −0.07	<0.001	603 (19.4)	615 (19.7)	0.01	−0.04, 0.06	0.73
Cerebrovascular disease	328 (6.0)	273 (6.4)	0.01	−0.04, 0.04	0.43	280 (9.0)	284 (9.1)	−0.04	−0.54, 0.45	0.90
Stroke	163 (3.0)	149 (3.5)	0.02	−0.01, 0.06	0.168	107 (3.4)	113 (3.6)	−0.01	−0.06, 0.04	0.73
Atrial fibrillation	501 (9.3)	437 (11.2)	0.06	0.02, 0.10	0.003	327 (10.5)	320 (10.3)	0.01	−0.04, 0.06	0.80
Urgent surgery	2155 (39.3)	1762 (41.5)	0.05	0.01, 0.09	0.030	1369 (43.9)	1389 (44.6)	−0.13	−0.65, 0.37	0.63

Data are presented as *n* (%) or mean (standard deviation).

After propensity score matching, 3116 patients from the ONCAB group were matched with 3116 patients from the OPCAB group. The ONCAB group had a significantly higher mean number of performed grafts, with a greater use of venous grafts, while the OPCAB group received more arterial and bilateral internal mammary artery (BIMA) grafts. There was no significant difference in the use or left internal mammary artery (LIMA) grafts between the groups (Table 2).

**Table 2:** Baseline characteristics of surgical details in all cohort and propensity-matched patients

Clinical variables	All patients					Propensity-matched patients				
	ONCAB (*n* = 5488)	OPCAB (*n* = 4432)	SMD	95% CI	*P*-value	ONCAB (*n* = 3116)	OPCAB (*n* = 3116)	SMD	95% CI	*P*-value
Number of performed grafts	2.73 (0.79)	2.11 (0.94)	0.72	0.68, 0.76	<0.001	2.69 (0.78)	2.12 (0.91)	0.68	0.63, 0.73	< 0.001
Arterial grafts	0.98 (0.50)	1.15 (0.57)	−0.33	−0.37, −0.29	<0.001	0.95 (0.47)	1.09 (0.52)	−0.28	−0.33, −0.23	<0.001
BIMA	158 (2.9)	199 (4.5)	−0.09	−0.13, −0.05	<0.001	90 (2.9)	155 (5.0)	−0.11	−0.16, −0.06	<0.001
LIMA	4303 (99.6)	381 (99.8)	−0.03	−0.07, 0.02	0.29	2446 (99.7)	2719 (99.9)	−0.04	−0.10, 0.02	0.29
Radial artery	69 (1.26)	130 (2.93)	−0.01	−0.16, −0.15	<0.001	39 (1.25)	99 (3.17)	−0.01	−0.21, −0.19	<0.001
Venous grafts	1.76 (0.83)	0.96 (0.87)	0.94	0.89, 0.98	<0.001	1.74 (0.85)	1.03 (0.90)	0.81	0.76, 0.87	<0.001

Data are presented as *n* (%) or mean (standard deviation).

In terms of postoperative outcomes, the ONCAB group demonstrated a significantly higher 30-day mortality rate (9.1% vs 6.4%, *P* = 0.002; OR 1.39). Additionally, the ONCAB group experienced a higher incidence of intra- or postoperative intraaortic balloon pump (IABP) use. Although not statistically significant, the ONCAB group also exhibited a higher rate of postoperative complications (Table 3). Moreover, the length of ICU stay was significantly longer in the ONCAB group (3.39(6.54) days vs 2.78(5.43) days, *P* < 0.001), as was the total length of hospital stay (10.15(10.26) days vs 9.59(10.12) days, *P* < 0.001). The mean follow-up time was similar between the groups, and no significant difference in overall mortality was observed (*P* = 0.28).

**Table 3:** Postoperative outcomes in all cohort and propensity-matched patients

Postoperative outcomes	All patients					Propensity-matched patients				
	ONCAB (*n* = 5488)	OPCAB (*n* = 4432)	OR	95% CI	*P*-value	ONCAB (*n* = 3116)	OPCAB (*n* = 3116)	OR	95% CI	*P*-value
30-day mortality	474 (8.6)	278 (6.3)	1.33	1.11, 1.58	0.002	284 (9.1)	198 (6.4)	1.39	1.13, 1.73	0.002
IABP intra- and postoperative	535 (9.7)	209 (4.7)	2.05	1.72, 2.43	<0.001	242 (7.8)	148 (4.7)	1.49	1.16, 1.90	<0.001
Intraoperative MI	82 (1.5)	57 (1.3)	1.04	0.73, 1.48	0.81	50 (1.6)	46 (1.5)	0.95	0.63, 1.45	0.83
Renal insufficiency	295 (5.4)	181 (4.1)	1.22	0.91, 1.64	0.173	184 (5.9)	140 (4.5)	1.18	0.89, 1.57	0.24
Postoperative dialysis	213 (3.9)	145 (3.3)	0.92	0.70, 1.20	0.55	129 (4.1)	112 (3.6)	0.83	0.61, 1.15	0.27
Gastrointestinal complications	72 (1.3)	45 (1.0)	1.07	0.72, 1.58	0.73	44 (1.4)	35 (1.1)	0.96	0.56, 1.63	0.87
Respiratory complications	386 (7.0)	240 (5.4)	1.20	0.99, 1.44	0.053	241 (7.7)	193 (6.2)	1.15	0.93, 1.43	0.20
Neurological complications	151 (2.8)	81 (1.8)	1.38	1.04, 1.83	0.024	91 (2.9)	63 (2.0)	1.32	0.94, 1.85	0.110
Multiorgan failure	149 (2.7)	91 (2.1)	0.82	0.55, 1.21	0.32	99 (3.2)	66 (2.1)	1.35	0.91, 2.02	0.137
Bleeding/tamponade	303 (5.5)	161 (3.6)	1.18	0.84, 1.65	0.33	172 (5.5)	124 (4.0)	1.16	0.75, 1.81	0.47
Rethoracotomy	316 (5.8)	165 (3.7)	1.29	0.92, 1.81	0.129	178 (5.7)	129 (4.1)	1.14	0.74, 1.76	0.56
Readmission to ICU	83 (1.5)	92 (2.1)	0.58	0.42, 0.79	<0.001	52 (1.7)	68 (2.2)	0.59	0.41, 0.87	0.008

Data are presented as *n* (%) or mean (standard deviation).

### Kaplan–Meier analysis

**Figure 2:**
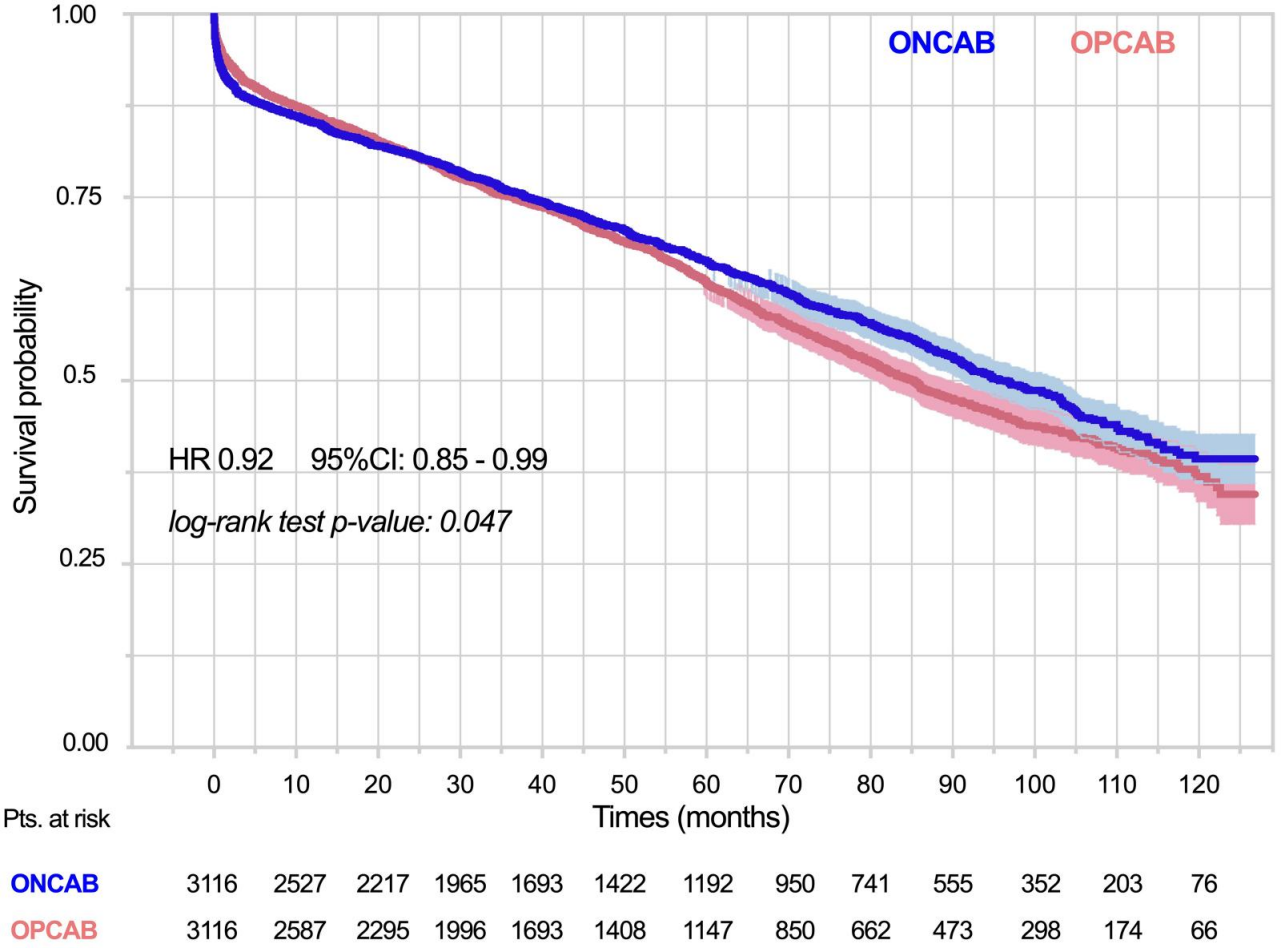
Kaplan–Meier survival curves, stratified by surgical strategy, in the matched patient cohort

Kaplan–Meier survival analysis revealed distinct trends over time for the OPCAB and ONCAB groups. At the 1-month mark, the survival rate in the OPCAB group was 94.6% (95% CI: 93.7–95.3), compared to 92.4% (95% CI: 91.6–93.4) in the ONCAB group. By 24 months post-surgery, the survival rates between the two groups had equalized. However, from this point onward, a gradual trend of improved survival emerged in the ONCAB group. At the 10-year follow-up, survival in the OPCAB group was 36.9% (95% CI: 31.1–38.7), while in the ONCAB group, it was 39.4% (95% CI: 36.9–42.3), with a statistically significant difference favouring ONCAB (*P* = 0.047) (Fig. 2).

## DISCUSSION

Over the 10-year study period, approximately 110 000 operations for isolated coronary artery disease were conducted in Poland, with 9% of these patients presenting with ICM. Roughly half of this population underwent OPCAB. The findings of this study contribute to the ongoing debate regarding the comparative outcomes of OPCAB and ONCAB in patients with severe left ventricular dysfunction. Despite the advancements in surgical techniques and perioperative care, our results indicate that ONCAB is associated with higher early postoperative risks compared to OPCAB, particularly in terms of 30-day mortality.

Over the years, CABG has shown long-term benefits in patients with ischaemic heart disease, outperforming conservative pharmacological therapy or PCI alone [8, 11, 12]. Early results are acceptable, but perioperative complications affect early survival. The STICH trial indicated that prolonged cardiopulmonary bypass time was the only surgical factor affecting in-hospital outcomes [4]. The choice of surgical strategy in ICM patients remains debated [13–17].

While some studies suggest advantages for OPCAB, recent research show increased risks of haemodynamic instability and urgent conversion to ONCAB in ICM patients [15, 17–20]. In our cohort, the conversion rate was low (2.5%), but the 30-day mortality rate among converted patients was high (25.5%). In contrast, Ueki *et al.* reported a higher conversion rate (6.1%) but a lower mortality rate (12.5%), while The Society of Thoracic Surgeons data indicated a 5.2% conversion rate with a further reduced mortality of 9.3% [16, 18]. The high mortality in converted patients may be due to haemodynamic instability during surgery.

Numerous studies have investigated the perioperative outcomes in ICM patients [13, 15–18]. OPCAB has been associated with lower incidence of in-hospital mortality, reoperation for bleeding, transfusion requirements, postoperative neurologic events, mediastinitis and prolonged mechanical ventilation [9, 17, 18]. OPCAB has been suggested to be safer in patients with renal insufficiency, potentially resulting in fewer cases of postoperative acute renal failure [21]. In our study, ONCAB was associated with higher 30-day mortality and more frequent readmissions to the ICU. However, long-term survival analysis revealed a slightly significant difference in favour of the ONCAB group at the 10-year follow-up. Zhou *et al.* [16] reported no significant difference in all-cause mortality, cardiovascular death or major adverse cardiovascular events between OPCAB and ONCAB over 10 years, despite an initial higher perioperative risk in the OPCAB group. Keeling *et al.* [17] found no differences in early mortality between the groups; however, after risk adjustment, in-hospital mortality was lower in the OPCAB group.

Takagi *et al.* [22] suggested that incomplete revascularization and worse graft patency may contribute to worse 5-year survival after OPCAB. Similarly, Kim *et al.* [23] reported worse long-term results after OPCAB. However, Carmona *et al.* [24] found better early outcomes in OPCAB patients, though no long-term survival benefit. It is important to note that none of these studies categorized patients based on LVEF. Major randomized trials, such as ROOBY and CORONARY, also failed to demonstrate consistent benefits for OPCAB [20, 24]. In the CORONARY trial, the composite outcome of death, stroke, myocardial infarction, renal failure or repeat revascularization at 5 years was similar between OPCAB and ONCAB groups [25]. However, the low number of high-risk ICM patients (<6%) in these studies complicates their analysis. Another meta-analysis showed early mortality reduction after OPCAB surgery in ICM patients, but this benefit did not last in the long term [15].

Our results indicate that OPCAB is associated with a lower 30-day mortality rate, despite a higher incidence of intraoperative conversion to ONCAB. The OPCAB group also experienced shorter ICU and hospital stays, though the ONCAB group tended to show better long-term survival.

### Study limitations

As a multicentre registry, the KROK database is not free of limitations related to invalid data entry. Despite continuous efforts to eliminate these shortcomings, adequate validation of the collected data is still necessary before the analysis. In addition, the propensity-matching analysis did not eliminate potential bias; however, this was partially compensated by a large dataset. Additionally, the learning curve implicating surgical experience and its impact on selection bias and outcomes was not assessed in this study. Moreover, data on left ventricular function were limited to LVEF parameters; other parameters such as left ventricular diameter of diastolic dysfunction that might have affected outcomes were not available. Although follow-up was achieved for all patients in our series, due to the verification of deaths in the national registry, we were unable to precisely compare mortality due to cardiovascular causes. Finally, the study lacked access to reintervention data, which could provide valuable insights into long-term survival outcomes.

## CONCLUSION

In conclusion, while OPCAB is associated with reduced early postoperative mortality, these short-term advantages do not translate into improved long-term survival. The choice between OPCAB and ONCAB should be individualized, taking into account both short-term and long-term outcomes, as well as factors such as patient-specific risks, comorbidities and surgical expertise. Tailoring the decision to each patient’s unique clinical profile is crucial for optimizing surgical outcomes.

## Data Availability

The data underlying this article will be shared on reasonable request to the corresponding author.

## ABBREVIATIONS

BIMA: Bilateral internal mammary artery
CABG: Coronary artery bypass grafting
CCS: Canadian Cardiovascular Society
IABP: Intra-aortic balloon pump
ICM: Ischaemic cardiomyopathy
ICU: Intensive care unit
LIMA: Left internal mammary artery
LVEF: Left ventricle ejection fraction
ONCAB: On-pump coronary artery bypass
OPCAB: Off-pump coronary artery bypass
PCI: Percutaneous coronary intervention
RA: Radial artery
